# Prevalence of high-risk HPV genotypes in sub-Saharan Africa according to HIV status: a 20-year systematic review

**DOI:** 10.4178/epih.e2021039

**Published:** 2021-05-25

**Authors:** Jude Ogechukwu Okoye, Chukwudi Amaechi Ofodile, Oluwaseun Kelechi Adeleke, Okechi Obioma

**Affiliations:** 1 Department of Medical Laboratory Science, Faculty of Health Sciences and Technology, Nnamdi Azikiwe University, Nnewi, Nigeria; 2 Department of Medical Laboratory Science, Afe Babalola University, Ado Ekiti, Nigeria; 3 Department of Medical Laboratory Science, Abia State University, Uturu, Nigeria

**Keywords:** Incidence, Viruses, Vaccines, Cervix Uteri, Africa

## Abstract

**OBJECTIVES:**

This review assessed the rate of high-risk human papillomavirus (HPV) infection among women living in sub-Saharan Africa. It also determined the prevalence of high-risk HPV (hrHPV) among human immunodeficiency virus (HIV) seropositive (HIV+) and seronegative (HIV-) women in sub-Saharan Africa, pre-2010 and post-2010.

**METHODS:**

In this systematic review, Google Scholar, PubMed Central, and Embase were searched to identify cohort and case-control studies that investigated the relationship between HIV and HPV infection. The database searches yielded 17 studies published between 1999 and 2018.

**RESULTS:**

In the general population, the prevalence of any HPV/multiple HPV infections was higher among HIV+ (53.6/22.6%) than among HIV- women (26.5/7.3%) with odds ratios of 3.22 and 3.71, respectively (95% confidence interval, 3.00 to 3.42 and 2.39 to 5.75, p<0.001). The prevalent HPV genotypes among HIV+ and HIV- women diagnosed with invasive cervical cancer (ICC) were HPV-16/18 and HPV-45. The prevalence of HPV-16, HPV-18, and HPV-45 was lower in 1999-2010 (3.8, 1.7, and 0.8%, respectively) than in 2011-2018 (19.1, 6.0, and 3.6%, respectively). Among women diagnosed with ICC, HIV+ women had a higher prevalence of HPV-56, HPV-31, and HPV-51 (7.3, 5.3, and 3.3%, respectively) than HIV- women (1.3, 2.2, and 0.4%, p<0.001, p=0.050, and p=0.013, respectively).

**CONCLUSIONS:**

The prevalence of HPV infection, multiple HPV infections, and non-vaccine HPV types were higher among HIV+ women than among HIV- women in sub-Saharan Africa. Although HIV infection influences the distribution of HPV types, this study suggests that cervical cancer incidence in sub-Saharan Africa is primarily driven by the prevalence of vaccine hrHPVs, especially HPV-16 and HPV-18.

## INTRODUCTION

Globally, cervical cancer is the third most common and deadly cancer among women [1]. The incidence of cervical cancer varies by race and region. According to the GLOBOCAN 2018 estimates, the mean age-standardized incidence rates (ASIR) for cervical cancer in sub-Saharan Africa and Northern Africa were 34.9 and 7.2, respectively [1,2]. This suggests that the ASIR of cervical cancer is higher in sub-Saharan Africa than in North Africa. Up to 2016, Jedy-Agba et al. [3] reported an increased incidence of cervical cancer in sub-Saharan Africa. The reason for the increasing incidence of cervical cancer in sub-Saharan Africa is still unknown. It could be related to the prevalence of human immunodeficiency virus (HIV) and human papillomavirus (HPV). In 2019, according to the Joint United Nations Program on HIV/AIDS, the population of people living with HIV in sub-Sahara Africa and Northern Africa was 12.9 million (3.9 to 23.0 million) and 240,000 (170,000 to 400,000), respectively [4]. Interestingly, the prevalence of people with HIV/AIDS accessing anti-retroviral therapy across sub-Sahara Africa ranges from 59.2% to 72.5% while that of Northern Africa was 38.3%. As of 2019, women and girls accounted for approximately 59% of those that were living with HIV in sub-Saharan Africa [4]. In sub-Saharan Africa, the prevalence of people living with HIV aged 15-49 years increased by 18.2% between 2000 and 2017 [5]. HIV facilitates HPV acquisition and delays its clearance with a concomitant increased risk of invasive cervical cancer (ICC) [6-9]. Belglaiaa et al. [10] maintained that HIV status is a strong predictor of high-risk HPV (hrHPV; odds ratio [OR], 4.16). Among HIV seropositive (HIV+) women, those who are positive for HIV-1/2 are 52% and 90% more like to be positive for hrHPVs than HIV-1+ and HIV-2+ women [11]. This suggests that the prevalence of HIV, especially HIV-1/2, may be responsible for the variation in the ASIR of cervical cancer between the African sub-regions. Additionally, a longer duration of HIV infection, higher viral load, and lower CD4 T-cell counts < 200/mm^3^ have also been implicated in a higher acquisition of HPV infection [12,13]. Considering race, in the United States, the incidence of HIV+ women diagnosed with cervical intraepithelial neoplasia grade 3 or higher was higher in African-Americans than Caucasians, with a ratio of 5:1. In the United States, the prevalent strains of hrHPV among HIV+ African-American women diagnosed with cervical cancer were HPV-16 (26.8%), HPV-53 (20.5%), HPV-35 (15.2%) and HPV-52/58 (14.3%) [14]. There is a paucity of data on the relationship between the types of HPV observed in African-American women and African women.

The introduction of national HPV immunization programs in countries in sub-Saharan Africa started in 2011 [2]. As of 2019, only 17 countries out of the 46 countries (37.0%) in sub-Saharan Africa had established nationwide HPV immunization [15]. Studies show that the HPV vaccine coverage is higher in Northern Africa (35.6%) than in sub-Saharan Africa (1.2%) [15,16]. The difference in vaccine coverage between the 2 regions may account for the higher ASIR of cervical cancer in the latter than the former. The prevalence of HPV infection could serve as an alternative index for assessing the impact of HPV vaccination on the risk of developing cancer. Widely distributed HPV vaccines targeted at reducing cervical cancer include bivalent (HPV-16/18) and quadrivalent (HPV6/11/16/18). The third, nonavalent vaccine (Gardasil 9; 6/11/16/18/31/33/45/52/58), has yet to be widely distributed in most African countries [17]. Sexually transmitted HPV genotypes are grouped into the high-risk type (HPV-16, -18, -31, -33, -35, -39, -45, -51, -52, -56, -58, -59, -66, and -68) and low-risk type (HPV-6, -11, -26, -40, -73, and -82) based on epidemiologic association and potential risk of cervical cancer [18-21]. Of note, individuals infected with multiple hrHPV genotypes are more likely to develop large tumors and have a poor treatment response [22], owing to a high propensity of co-existing with other hrHPVs than low-risk HPVs [23]. The co-existence of the non-vaccine hrHPV reduces the efficacy of vaccines in preventing cervical cancer. According to Yar et al. [23], the involvement of non-vaccine hrHPV in hrHPV co-infections among African women was higher for HPV-35 (19.6%), followed by HPV-53 (15.0%), HPV-56 (7.5%), HPV-59/66 (6.5%), and HPV-82 (5.6%). Since the introduction of HPV vaccination into the national immunization program, to the best of our knowledge, no study has assessed the prevalence of HPV types, especially among HIV+ and HIV seronegative (HIV-) women, in sub-Saharan Africa between years up to 2010 and in 2011 and later; hence, the present review was conducted to address this gap in the research. This review suggests that a high prevalence of non-vaccine hrHPV and multiple HPV infections could be associated with the high ASIR of cervical cancer in sub-Saharan Africa.

## MATERIALS AND METHODS

This systematic review was carried out (up to September 16, 2020) in accordance with PRISMA (Preferred Reporting Items for Systematic Reviews and Meta-Analyses) guidelines [24,25].

### Search strategy

**Figure 1.**
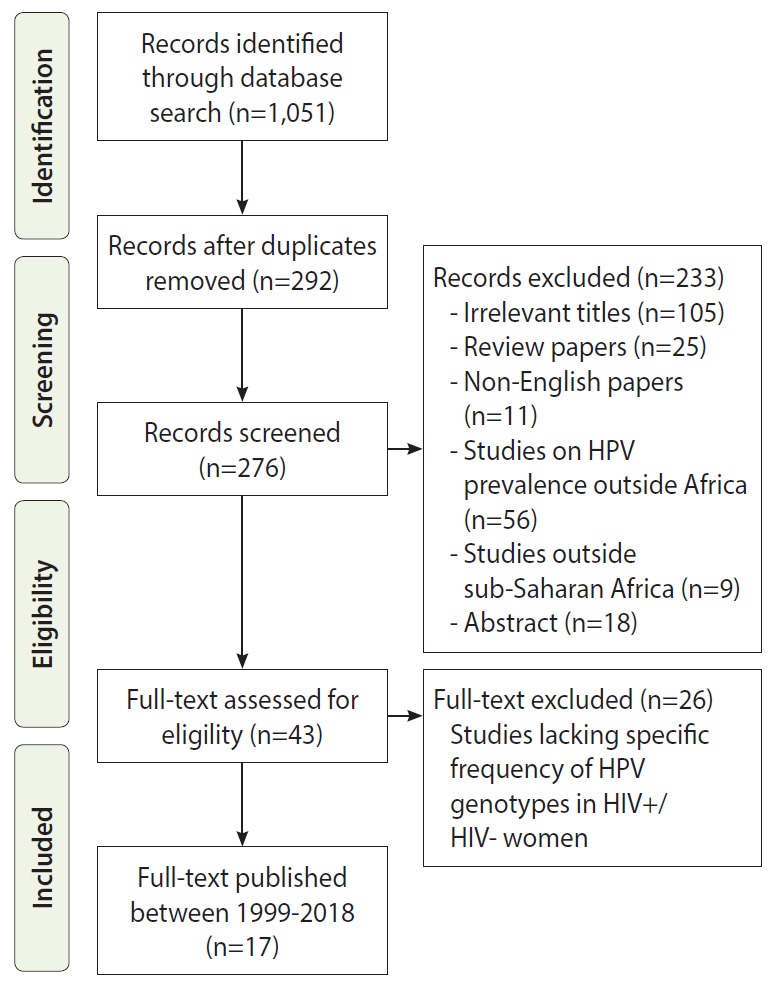
PRISMA (Preferred Reporting Items for Systematic Reviews and Meta-Analyses) flow diagram for studies on the prevalence of human papillomavirus (HPV) among women living seropositive (HIV+) and seronegative (HIV-) human immunodeficiency virus (HIV) in sub-Saharan Africa.

Studies that investigated the relationship between HIV status and HPV infection or acquisition were searched for in Google Scholar, Scopus, PubMed Central, and Embase databases and selected using the PRISMA guidelines (Figure 1). We screened the titles of cohort and case-controlled studies published between 1999 and 2018 using the following keywords and Medical Subject Headings (MeSH) terms: (‘HPV’ and ‘human papillomavirus’) AND (‘HIV’ and ‘human immunodeficiency virus’) AND (‘ICC’ and ‘invasive cervical cancer’) AND (‘prevalence’ OR ‘incidence’ OR ‘distribution’ OR ‘genotype’), AND (‘sub-Saharan Africa’). We also searched for unpublished studies (gray literature) by evaluating ClinicalTrials.gov (National Institutes of Health, NIH) and the International Clinical Trial Registry Platform (World Health Organization, WHO).

### Study quality assessment and study selection

The quality of the included studies was assessed using an adapted version of the NIH’s Quality Assessment Tool for Observational Cohort and Cross-Sectional Studies [26]. Three authors assessed the risk of bias (as good, unclear, or poor) in non-randomized observational studies across 5 criteria: study population, imprecision, inconsistency, bias in study design, and disclosure of conflict of interest [27,28]. The exclusion criteria were articles not written in English, abstracts, non-full-length articles, articles without specific frequency of HPV types, articles not involving Africa, and articles not dealing with cervical cancer. The inclusion criteria were studies that tested for hrHPV DNA, studies with specific frequency of hrHPV infection, and full-length articles involving sub-Saharan Africa.

**Figure 2.**
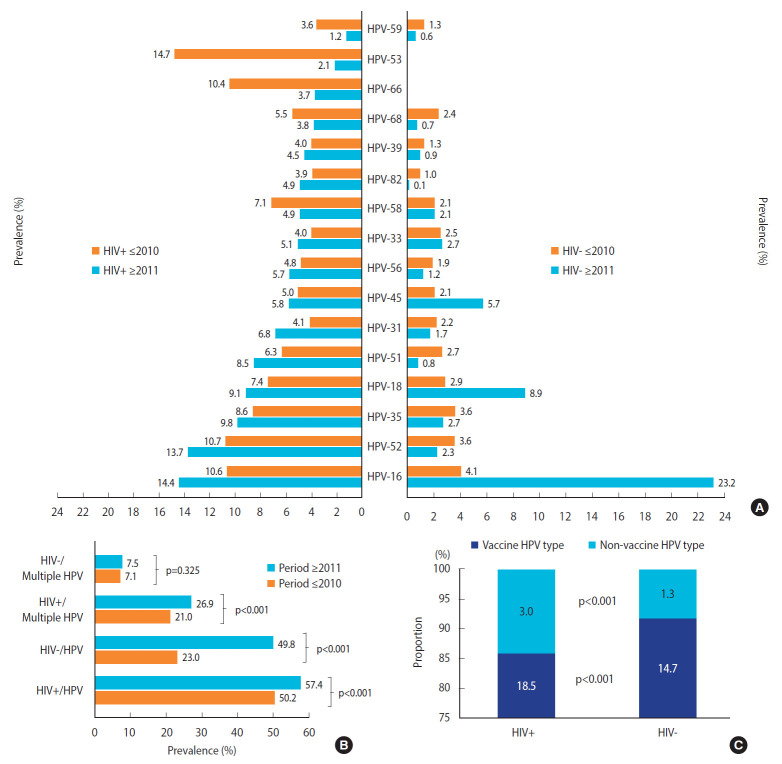
Prevalence of any human papillomavirus (HPV) infection (A) and multiple HPV infections (B), as well as vaccine and non-vaccine HPV types (C), in women living seropositive (HiV+) and seronegative (HIV-) human immunodeficiency virus (HIV) from 1999 to 2018 in subSaharan Africa (general population).

Only studies that used polymerase chain reaction, which is the gold standard for HPV testing, were included in this study (Tables 1-3). The overall prevalence of an HPV type (for example HPV-16) was dependent on the study size of studies that tested participants for the specific virus. In Figure 2A and B, the study of Menon et al. [19], which was carried out between 2009 and 2015, was excluded because its data cut across the 2 time periods (up to 2010 and in 2011 and later). The studies carried out by Diop-Ndiaye et al. [29], Dols et al. [30] and Denny et al. [31] were excluded from the calculations of the prevalence of multiple HPV infections due to lack of data (Table 4). The mean age of HIV+ and HIV- women did not include data from Mpunga et al. [32], Mudini et al. [20], Marembo et al. [33], Maranga et al. [34], and Banura et al. [35] because they did not specify the mean age and age range of the 2 groups. The data presented in Table 5 were only extracted from the studies carried out by Mpunga et al. [32], Mudini et al. [20], and Maranga et al. [34] due to the fact that their papers reported the prevalence of HPV types for both HIV+ and HIV- women diagnosed with cancer.

### Data extraction

The essential information extracted for analysis included participant characteristics such as sample size, cases of HIV+ and HIV- women, the prevalence of any HPV infection and multiple HPV infections, HPV types (16, 18, 31, 33, 35, 39, 45, 51, 52, 53, 56, 58, 59, 66, 68, and 82), mean age, recruitment method, period of data collection, and study location and region (according to the WHO classification). We investigated the frequency of HPV infections in women living with HIV using HIV- women as the comparison group. When calculating the prevalence of any HPV infection (women who tested positive for any HPV type), an individual may have acquired multiple types (e.g., HPV-35 and -45) but it would only count as 1 event. The range of high-risk HPVs investigated in the selected studies varied; thus when calculating the prevalence of an HPV type, only studies or cases that investigated that particular HPV type were considered. To assess the impact of the time period on the prevalence of HPV infection, data points were categorized into a group until 2010 (inclusive) and a post-2010 group.

### Statistical analysis

The ORs between HIV+ and HIV- women were also calculated in order to determine the risk of HPV acquisition and development of cervical cancer. Chi-square analysis was used to calculate the difference in HPV infection between HIV+ and HIV- women in Africa (GraphPad Prism version 6.0; GraphPad, San Diego, CA, USA), and the level of statistical significance was set at p-value ≤ 0.05.

### Ethics statement

This review is exempt from ethical review and approval, since the secondary data used for pooled analysis were extracted from journal-related publications.

## RESULTS

### Selected studies and sample size

Based on the inclusion criteria, 11 cross-sectional studies and 6 cohort studies were analyzed. Overall, this review included 16,237 participants (N) from 17 full-length articles (Figure 1 and Table 1). The number of HIV+ and HIV- women were 5,341 and 10,896, respectively. Southern Africa had the highest number of participants (10,285; n=3 studies), followed by West Africa (3,553; n=9 studies), and East Africa (2,399; n=5 studies), and Southern Africa (10,285; n=3 studies). The mean age of HIV- women was insignificantly higher than that of HIV+ women (38.1 vs. 36.2 years, p=0.59). The prevalence of HPV infection and multiple hrHPV infections in the cohort studies (which involved HIV+ women only) were twice the prevalence in the cross-sectional studies (which included both HIV+ and HIV- women). No cohort studies involving only HIV- women were identified. The 3 most prevalent HPV types in the cohort studies were HPV-52, HPV-16, and HPV-35, while those in the cross-sectional studies were HPV-16, HPV-18, and HPV-35 (Table 2). Table 3 shows the summary of the findings from each study.

### Prevalence of human papillomavirus (HPV) and multiple HPV infections in the general population

The prevalence of various HPV types and multiple HPV infections was higher in HIV+ women than in HIV-women (p<0.001) (Table 4). The most prevalent HPV type in the general population of sub-Saharan Africa was HPV-16, followed in order by HPV-66, HPV-53, and HPV-52. Furthermore, HIV+ women in subSahara Africa were approximately 3 times and 4 times more likely to be HPV-infected and to have multiple HPV infections, respectively (Table 4). Table 4 also shows that HIV+ women were approximately 2 times, 3 times, 4 times, and 5 times more likely to acquire HPV-16/-18/-33/-45/-53/-59, HPV-31/-35/-56/-58/-68, HPV-39/-52, and HPV-82, respectively than their HIV- counterparts. HIV+ women were 26% more likely to acquire HPV-66 than HIV- women.

### Human papillomavirus infection and study timing

Between 1999-2010 and 2011-2018, the prevalence of HPV-16, HPV-18, and HPV-45 among HIV- and HIV+ women increased by 19.1%p versus 3.8%p, 6.0%p versus 1.7%p, and 3.6%p versus 0.8%p, respectively (Figure 2). Furthermore, the prevalence of HPV-53, HPV-66, HPV-59, HPV-58, and HPV-68 decreased by 12.6%p, 6.7%p, 2.4%p, 2.2%p, and 1.7%p among HIV+ women, respectively. Overall, the prevalence of HPV and multiple HPV infections increased in sub-Sahara Africa within the study period (Figure 2).

As shown in Figure 2A, in sub-Saharan Africa, the prevalence of HPV types among HIV- women up to 2010 was higher than in 2011 and later, except for HPV-16, HPV-18, and HPV-45. Furthermore, the prevalence of HPV types among HIV+ women up to 2010 was lower than in 2011 and later, except for HPV-58, HPV68, HPV-66, HPV-53, and HPV-59. A higher prevalence of HPV infection was observed in 2011 and later than up to 2010 both in HIV+ and HIV- women (Figure 2B). The difference in the prevalence of HPV infection, the difference between 2011 and later and up to 2010 was higher among HIV- women than in HIV+ women (26.8 vs. 7.2%). Among HIV- women, the prevalence of HPV infection doubled between 2010 and 2018, unlike HPV prevalence in HIV+ women, which increased by 5.4%p (Figure 2B). Furthermore, a higher prevalence of multiple HPV infections was observed in 2011 and later than up to 2010 both in HIV+ women (p<0.05) and HIV- women (p>0.05). In Figure 2C, a higher prevalence of vaccine and non-vaccine HPV types were observed among HIV+ women when compared with their HIV- counterparts (18.5 vs. 14.7%, and 3.0 vs. 1.3%, respectively at p<0.001). However, a higher frequency of vaccine HPV types was observed among HIV- women than among HIV+ women (91.9 vs. 86.0%), whereas a higher frequency of non-vaccine HPV types was observed among HIV+ women than among HIV- women (14.0 vs. 8.1%) at p<0.001.

### Prevalence of multiple infections and human papillomavirus types among women with cervical cancer

Among women diagnosed with ICC, the prevalence of multiple HPV infections was higher in HIV+ women than in HIV- women (28.9 vs. 13.1%, respectively; p<0.001). In descending order, the most prevalent hrHPV types among HIV+ and HIV- women diagnosed with ICC were HPV-16 (51.9 vs. 58.2%), HPV-18 (24.6 vs. 18.5%), and HPV-45 (12.8 vs. 12.6%, respectively). Significant differences between HIV+ and HIV- women diagnosed with ICC were only observed for the prevalence of HPV-56 (7.3 vs. 1.3%), HPV-31 (5.3 vs. 2.2%), and HPV-51 (3.3 vs. 0.4%, respectively) at p<0.001, p=0.050, and p=0.013, respectively. HIV+ women in sub-Saharan Africa who were positive for HPV-56/51 and HPV-68 were 6 times and 5 times more likely to develop cervical cancer than their HIV- counterparts (p<0.001/p=0.013 and p=0.125, respectively). Figure 2 also shows that HIV+ women with multiple HPV infections and HPV-31/-39/-58 were approximately 3 times more likely to develop cervical cancer than HIV- women. Furthermore, HIV+ women with HPV-16 and HPV-35 were 23% and 51% less likely to develop cervical cancer than their HIV- counterparts (Table 5). As shown in Table 5, the prevalence of all HPV types, including multiple HPV types, was higher among HIV+ women than among HIV- women, except HPV-16 and HPV-35.

## DISCUSSION

This study assessed the prevalence of HPV types among HIV+ and HIV- women in regions of sub-Saharan Africa and between 2 time intervals: up to 2010 and 2011 and later. The prevalence of HPV and multiple HPV infections was higher among HIV+ women than among HIV- women living in sub-Saharan Africa. Between 2013 and 2016, this pattern of infection was also observed among HIV+ and HIV- women in North Africa (65.7 vs. 13.3% and 38.5 vs. 7.6% respectively) [10,13]. The lower ASIR of cervical cancer in North Africa could be attributed to higher HPV vaccine coverage than in sub-Saharan Africa [16]. Conversely, the reason(s) for the differences between HIV+ and HIV- women are not well-understood. However, previous studies [11,42] opined that HIV+ women initiate sex at a younger age, and therefore have a higher number of lifetime sexual partners, in turn increasing their risk of acquisition and persistence of HPV infections relative to their HIV- counterparts. As of 2016, the review carried out by Clifford et al. [8] shows that HPV-16 (46.6%), HPV-18 (24.4%), and HPV-45 (15.5%) were the prevalent HPV types in HIV+ women diagnosed with ICC in Africa. Our findings further show that HPV-16, HPV-18, and HPV-45 were the prevalent HPV types not only among HIV+ women, but also among HIV- women in sub-Saharan Africa. The prevalence of HPV-16 and HPV-18 among HIV+ women in this review were higher than that of Clifford et al. [8], possibly due to differences in study timelines and regions involved [11]. The differences observed between HIV+ and HIV- women suggest that the prevalence of HIV in a population increases the risk of acquiring HPV and multiple infections [20,29,34]. These factors, in turn, predict a disproportionate ASIR of cervical cancer among HIV+ and HIV- women in sub-regions of Africa [19]. Additionally, in sub-Saharan Africa, the prevalence of HPV-16 and HPV-35 infections was higher in HIV- women diagnosed with ICC than in their HIV+ counterparts. The reason for this is unknown.

Studies have shown that HPV-infected women, especially those infected with hrHPV types, are approximately at a 2-fold higher risk of acquiring HIV than HPV-uninfected women [43,44]. A follow-up investigation carried out among HIV-uninfected women showed that 28.4% of HPV-infected women seroconverted after an average of 2.4 years. The study revealed that women with multiple hrHPV infections were 4 times more likely to acquire HIV than those with a single or no hrHPV infection [45]. The high-risk of developing cervical cancer among HIV+ women and the risk of seroconversion among HIV- women due to the high prevalence of multiple HPV infections may account for the high ASIR of cervical cancer in sub-Saharan Africa [1]. According to Smith-McCune et al. [46], women with nonavalent vaccine types are 2.5 times more likely to acquire HIV than women with vaccine HPV types. The high ASIR of cervical cancer in sub-Saharan Africa, when compared to North Africa may be due to a higher prevalence of nonavalent hrHPV among HIV+ and HIV- women. Of note, the most prevalent HPV types among HIV+ women in North Africa were vaccine HPV types: HPV-58 (22.1%), HPV-18 (7.8%), HPV-16 (7.3%), HPV-33 (6.0%), and HPV-52 (3.7%) [10,13]. Conversely, in order of prevalence, the most prevalent HPV types among people living with HIV in sub-Saharan Africa irrespective of cytology status were HPV-16, HPV-52, HPV-53, HPV-35, and HPV-66. This suggests that with the available vaccine, it could be easier to reduce or prevent cervical cancer attributable to HPV in North Africa than in sub-Saharan Africa. Taken together, HIV+ women in sub-Saharan Africa are at a higher risk of developing cervical cancer despite the available vaccine due to a higher prevalence of non-vaccine hrHPV types. Since women who are HPV-16 and HPV-18-positive are 11-22 times and 45-58 times capable of acquiring other hrHPV types, respectively [47], it could be argued that the lower prevalence of non-vaccine HPV types among HIV+ women in North Africa, when compared with their sub-Sahara African counterparts, was due to the low prevalence of HPV-16 and HPV-18 (Table 4).

This study revealed that the prevalence of HPV-16, HPV-18, and HPV-45 increased from the time period up to 2010 to 2011 and later in both HIV+ and HIV- women. The increase could be due to increasing awareness of cervical cancer and uptake of screening services, better screening and testing techniques or protocols, or changes in policy. Interestingly, the percentage difference in HPV infection between up to 2010 and 2011 and later was considerably higher in HIV- women. The reason for this is also unknown. Similarly, the changes in the prevalence of HPV-16 between both time periods among HIV+ and HIV- women were significant (p<0.001), but substantially higher among HIV-women than in HIV+ women (Figure 1). This agrees with the findings of Dames et al. [12] showing lower HPV infection among women with CD4+ T-cell counts of ≥ 200 cells/mm^3^. On the other hand, increased sexual behavior or activity and a higher frequency of unprotected sex may be responsible for the marked increase in HPV prevalence among HIV- women in sub-Saharan Africa. Low CD4+ T-cell counts, lower age, a history of multiple sexual partners, and a high number of unprotected sexual intercourse, especially among infected persons living with HIV [13,31] may also be responsible for multiple HPV infections among HIV+ women.

Based on the database search, studies from Central Africa did not meet the inclusion criteria. Since Central Africa is a sub-region of sub-Saharan Africa, the non-inclusion of studies from the sub-region constitutes a limitation of this study. Moreover, in this study, a meta-analysis was not carried out; hence, there could be possibility of some publication bias or heterogeneity.

## CONCLUSION

This paper reveals that the prevalence of HPV infection, multiple HPV infections, and non-vaccine HPV types were higher among HIV+ women than in HIV- women in sub-Saharan Africa. This paper revealed that the prevalence of hrHPV, especially HPV-16 and HPV-18, increased over the last decade irrespective of HIV status. Although HIV infection influences the distribution of HPV types, this study suggests that cervical cancer incidence in sub-Saharan Africa is primarily driven by the prevalence of vaccine hrHPVs, especially HPV-16 and HPV-18.

## Figures and Tables

**Table 1.** Findings of selected studies in sub-Saharan Africa on HPV infection according to HIV status

Study		Location	Period of study	Mean age (range), yr	Study size	Multiple HPV (%)	Order of 6 most prevalent high-risk HPV types
Studies of HIV seropositive women							
	Mpunga et al. [32]^1^	Rwanda	2012-2018	54.3 (NA)^4^	99	7 (7.2)	16, 35, 45, 31, 33, 52
	Yakub et al. [18]	Nigeria	2016-2017	NA (20-50)	220	25 (11.4)	35, 16, 45, 33, 18, 56
	Ndizeye et al. [36]	Burundi	2013/2016	39.9 (NA)	301	28 (9.3)	16, 18, 51, 52, 58, 56/66
	Mudini et al. [20]^1^	Zimbabwe	2014-2015	NA (40-60)^4^	53	30 (56.6)	16, 18, 56, 45, 33, 58
	Obiri-Yeboah et al. [37]	Ghana	2017	43.8 (NA)	160	77 (48.1)	35, 52, 58, 16, 18, 68
	Marembo et al. [33]	Zimbabwe	2015	39.8 (18-83)^4^	70	17 (24.3)	52, 16, 18, 58, 51, 31/33/45
	Menon et al. [19]^2^	Kenya	2009-2015	28.0 (NA)	74	48 (64.9)	16, 53, 52, 56, 18/35/58,
	Ezechi et al. [38]	Nigeria	2014	NA (NA)	220	18 (8.2)	16, 35, 31, 58, 52, 18/45
	Akarolo-Anthony et al. [21]	Nigeria	2012	36.6 (NA)	149	21 (14.1)	35, 56, 58, 59, 45, 33
	Kelly et al. [39]	SA	2011-2012	NA (20-50)	594	147 (24.7)	52, 51, 35, 16, 31, 39
	Kelly et al. [39]	Burkina Faso	2011-2012	NA (20-50)	621	271 (43.6)	52, 16, 35, 51, 18, 31
	Diop-Ndiaye et al. [29]^3^	Senegal	2010	36.0 (30-45)	67	NA	52, 16, 68, 35, 45, 51
	Dols et al. [30]	Tanzania/SA	2008-2010	NA (NA)	194	NA	52, 16, 51, 35, 58, 18
	Guthrie et al. [40]	Kenya	2007-2009	NA (18-50)	283	122 (43.1)	52, 18, 16, 51, 35, 68
	Maranga et al. [34]	Kenya	2008-2009	35.3 (21-50)^4^	113	22 (19.5)	52, 56, 58, 53, 16, 35/39/66
	McDonald et al. [41]	SA	1999-2006	NA (26-38)	1641	249 (15.2)	35, 16, 58, 18, 68, 45
	Banura et al. [35]	Uganda	2002-2004	NA (12-24)^4^	82	53 (64.6)	52, 33, 16, 51, 68, 66
	Denny et al. [31]	SA	2000-2003	29.3 (18-54)	400	NA	16, 52, 53, 35, 18, 58
Studies of HIV seronegative women							
	Mpunga et al. [32]^1^	Rwanda	2012-2018	54.3 (NA)^4^	501	21 (4.2)	16, 18, 45, 33, 35, 52
	Ndizeye et al. [36]	Burundi	2013/2016	36.4 (NA)	299	9 (3.0)	16, 18, 66, 45, 58, 53
	Mudini et al. [20]^1^	Zimbabwe	2014-2015	NA (40-60)^4^	54	25 (46.3)	16, 13, 33, 35, 56, 45
	Obiri-Yeboah et al. [37]	Ghana	2017	44.3 (NA)	169	36 (21.3)	35, 33, 58, 56, 52, 18/39/68
	Marembo et al. [33]	Zimbabwe	2015	39.8 (18-83)^4^	66	10 (15.2)	18, 16, 52, 31, 45/51/58
	Ezechi et al. [38]	Nigeria	2014	NA (NA)	295	10 (3.4)	18, 58, 16, 52, 31/35/51
	Akarolo-Anthony et al. [21]	Nigeria	2012	37.6 (NA)	108	2 (1.9)	52, 68, 18, 39, 45, 16/31/56/59
	Diop-Ndiaye et al. [29]^2^	Senegal	2010	34.0 (26-42)	369	NA	52, 64, 16, 51, 35, 31/33
	Maranga et al. [34]	Kenya	2008-2009	35.3 (21-50)^4^	111	15 (13.5)	56, 16, 33, 35, 59, 51/52/82
	McDonald et al. [41]	SA	1999-2006	NA (33-45)	8,050	301 (3.7)	35, 16, 58, 45, 52, 18
	Banura et al. [35]	Uganda	2002-2004	NA (12-24)^4^	868	324 (37.3)	18, 52, 16, 51, 33, 68

HPV, human papillomavirus; HIV, human immunodeficiency virus; NA, not available; SA, South Africa; FSW, female sex workers.

1 Cancer.

2 FSW with abnormal cytology.

3 FSW.

4 Age of all participants (HIV seropositive and HIV seronegative).

**Table 2.** Findings of selected studies in sub-Saharan Africa based on human immunodeficiency virus status

Study		Countries	Study period	Cases, n	High-risk HPV (%)	Multiple-HPV prevalence (%)	Order of 6 most prevalent high-risk HPV types
Cross-sectional studies (HIV+/HIV-)							
	Mpunga et al. [32]^1^	Rwanda	2012-2018	598	574 (96.0)	28 (4.7)	16, 18, 45, 33, 35, 52
	Mudini et al. [20]^1^	Zimbabwe	2014-2015	107	101 (94.4)	55 (51.4)	16, 18, 56, 33, 45, 35
	Banura et al. [35]	Uganda	2002-2004	950	707 (74.4)	377 (39.7)	52,16, 18, 51, 33, 56
	Diop-Ndiaye et al. [29]^2^	Senegal	2010	436	316 (72.5)	NA	52, 16, 68, 35, 51, 33
	Marembo et al. [33]	Zimbabwe	2015	136	70 (51.5)	27 (19.9)	18, 52, 16, 58, 51, 31
	Obiri-Yeboah et al. [37]	Ghana	2017	329	156 (47.4)	113 (34.3)	35, 58, 52, 18, 56, 16/56
	Maranga et al. [34]	Kenya	2008-2009	224	105 (46.9)	37 (16.5)	56, 52, 58, 16, 35, 33
	Akarolo-Anthony et al. [21]	Nigeria	2012	257	64 (24.9)	23 (8.9)	82, 35, 56, 58, 45, 59
	Ndizeye et al. [36]	Burundi	2013/2016	600	142 (23.7)	37 (6.2)	16, 18, 58, 52, 51, 31
	Ezechi et al. [38]	Nigeria	2014	515	101 (19.6)	28 (5.4)	16, 35, 58, 31, 18, 52
	McDonald et al. [41]	SA	1999-2006	9,691	1,848 (19.1)	550 (5.7)	35, 16, 58, 45, 18, 52
	Total [20, 21, 29, 32-38, 41]	Africa	1999-2018	13,843	4,184 (30.2)	1,275 (9.5)	16, 18, 35, 52, 45, 58
Cohort studies (HIV+ only)							
	Yakub et al. [18]	Nigeria	2016-2017	220	83 (37.7)	25 (11.4)	35, 16, 45, 33, 18, 56
	Menon et al. [19]^3^	Kenya	2009-2015	74	52 (70.2)	48 (64.9)	16, 53, 52, 56, 18/35/58
	Kelly et al. [39]	Burkina Faso	2011-2012	621	491 (79.1)	271 (43.6)	52, 16, 35, 51, 18, 31
	Kelly et al. [39]	SA	2011-2012	594	351 (59.1)	147 (24.7)	52, 51, 35, 16, 31, 39
	Dols et al. [30]	Tanzania/SA	2008-2010	194	109 (56.2)	NA	52, 16, 51, 35, 58, 18
	Guthrie et al. [40]	Kenya	2007-2009	283	176 (62.2)	122 (43.1)	52, 18, 16, 51, 35, 68
	Denny et al. [31]	SA	2000-2003	400	301 (75.3)	NA	16, 52, 53, 35, 18, 58
	Total [18, 19, 30, 31, 39, 40]	Africa	2000-2017	2,386	1,563 (65.5)	613 (34.2)	52, 16, 35, 18, 51, 31

HPV, human papillomavirus; HIV+, human immunodeficiency virus seropositive; HIV-, human immunodeficiency virus seronegative; NA, not available; SA, South Africa; FSW, female sex workers.

1 Cancer,

2 FSW.

3 FSW with abnormal cytology.

**Table 3.** Findings of selected studies in sub-Saharan Africa based on HIV status

Study	Location	Summary/Inferences
Mpunga et al. [32]^1^	Rwanda	There was a minimal impact of HIV on HPV type distribution
Yakub et al. [18]	Nigeria	HIV+ women with a low CD4+ T count were at a higher risk of cervical precancerous lesions
Ndizeye et al. [36]	Burundi	There was a high burden of hrHPV and phrHPV infections among women with HIV; The nonavalent vaccine covered most of the hrHPV infections irrespective of residential area and HIV status
Mudini et al. [20]^1^	Zimbabwe	HIV may inﬂuence the distribution of some HPV genotypes given the signiﬁcant increase in prevalence of HPV-18 among HIV+ women; The proportion of women with multiple genotypes was high and almost equal in both HIV+ and HIV- women
Obiri-Yeboah et al. [37]	Ghana	HIV-1 infected women bore a significant burden of HPV infection and related disease; The nonavalent HPV vaccine is likely the best means of cervical cancer prevention in Ghana
Marembo et al. [33]	Zimbabwe	There was an increased risk of hrHPV infection as well as multiple hrHPV genotypes in HIV+ women
Menon et al. [19]^2^	Kenya	Co-infection with phrHPV and hrHPV genotypes was more strongly associated with abnormal cytology than any single hrHPV; There was a high prevalence of multiple hrHPV genotypes in FSW, especially in HIV+ women
Ezechi et al. [38]	Nigeria	HPV-16, -35, -58, and -31 were the most common hrHPV infections in the population and HIV+ women awere at higher risk of acquiring HPV infection; Current HPV vaccines prevented genotypes 16 and 18, which accounted for only a minority of hrHPV infection (21.7%) with no significant difference been HIV+ and HIV- women
Akarolo-Anthony et al. [21]	Nigeria	There was a high prevalence of non-16 and -18 hrHPV among HIV+ women in Nigeria and other African countries
Kelly et al. [39]	SA/Burkina Faso	hrHPV infections and cervical lesions were very common among HIV+ women in Africa; Bivalent or quadrivalent vaccines could prevent up to 45% of treatable precursor lesions, and the nonavalent vaccine could prevent up to 90% of cases in HIV+ women
Diop-Ndiaye et al. [29]^3^	Senegal	HPV-16 and -35 were the most prevalent HPV types among HIV-infected FSW
Dols et al. [30]	Tanzania/SA	More than one-third (42%) of women with normal cytology tested positive for hrHPV
Guthrie et al. [40]	Kenya	hrHPV prevalence was high in HIV+ women; Screening for hrHPV genotypes would identify a large majority of women who have high-grade cervical lesions or more severe cytology
Maranga et al. [34]	Kenya	HIV infection appeared to alter the spectrum of HPV types found in both cervical smears and invasive cervical carcinomas; HPV infections were associated with a reduced level of immunity
McDonald et al. [41]	SA	HPV-16 and -35 were the prevalent HPV types among HIV+ and HIV- women with or without cervical disease
Banura et al. [35]	Uganda	There was an elevated prevalence of HPV infection in HIV+ and HIV- young women
Denny et al. [31]	SA	There was a high level of hrHPV infections in HIV-1 infected women

HIV, human immunodeficiency virus; HPV, human papillomavirus; hrHPV, high-risk human papillomavirus; phrHPV, probable high-risk human papillomavirus; HIV+, human immunodeficiency virus seropositive; HIV-, human immunodeficiency virus seronegative; SA, South Africa; FSW, female sex workers.

1 Cancer.

2 FSW with abnormal cytology.

3 FSW.

**Table 4.** Prevalence of high-risk HPV types in sub-Saharan Africa based on HIV status

HPV type	Total population		HIV seropositive		HIV seronegative		% Diff (n1-n2)	OR (95% CI)
	Cases, N	HPV, n (%)	Cases, N	HPV n1 (%)	Cases, N	HPV n2 (%)		
Any HPV	16,237	5,747 (35.4)	5,341	2,865 (53.6)	10,896	2,882 (26.5)	27.1	3.22 (3.00, 3.42)
Multiple	15,001	1,860 (12.4)	4,981	1,128 (22.6)	10,020	732 (7.3)	15.3	3.71 (2.39, 5.75)
HPV-16	16,237	1,404 (8.6)	5,341	673 (12.6)	10,896	731 (6.7)	5.9	2.00 (1.67, 2.39)
HPV-18	16,237	854 (5.2)	5,341	450 (8.4)	10,896	404 (3.7)	4.7	2.39 (2.36, 2.41)
HPV-31	16,237	526 (3.2)	5,341	297 (5.6)	10,896	229 (2.1)	3.5	2.74 (2.32, 3.25)
HPV-33	16,237	531 (3.3)	5,341	255 (4.8)	10,896	276 (2.5)	2.3	1.93 (1.63, 2.29)
HPV-35	16,237	876 (5.4)	5,341	501 (9.4)	10,896	375 (3.4)	6.0	2.90 (2.69, 3.10)
HPV-39	16,237	366 (2.3)	5,341	235 (4.4)	10,896	131 (1.2)	3.2	3.78 (3.03, 4.71)
HPV-45	16,237	580 (3.6)	5,341	295 (5.5)	10,896	285 (2.6)	2.9	2.19 (1.93, 2.46)
HPV-51	16,124	653 (4.0)	5,228	389 (7.4)	10,896	264 (2.4)	5.0	3.23 (2.75, 3.78)
HPV-52	16,237	1,018 (6.3)	5,341	644 (12.1)	10,896	374 (3.4)	8.7	3.86 (3.60, 5.86)
HPV-53	2,528	174 (6.9)	881	90 (10.2)	1,647	84 (5.1)	5.1	2.12 (1.80, 2.48)
HPV-56	16,237	494 (3.0)	5,341	296 (5.5)	10,896	198 (1.8)	3.7	3.17 (2.61, 3.82)
HPV-58	16,237	561 (3.5)	5,341	335 (6.3)	10,896	226 (2.1)	4.2	3.16 (2.66, 3.76)
HPV-59	16,237	263 (1.6)	5,341	135 (2.5)	10,896	128 (1.2)	1.3	2.18 (1.70, 2.80)
HPV-66	2,903	225 (7.8)	1,202	109 (9.1)	1,701	116 (6.8)	2.3	1.26 (1.09, 1.45)
HPV-68	16,237	471 (2.9)	5,341	257 (4.8)	10,896	214 (2.0)	2.8	2.52 (2.10, 3.00)
HPV-82	1,241	47 (3.8)	707	40 (5.7)	534	7 (1.3)	4.4	4.51 (3.00, 6.82)

HPV, human papillomavirus; HIV, human immunodeficiency virus; Diff, difference; OR, odds ratio; CI, confidence interval; N, number of cases inves tigated (with adequate information on HPV positivity).

**Table 5.** Prevalence of high-risk HPV types in invasive cervical cancer patients based on HIV status

HPV type	HIV seropositive		HIV seronegative		% Diff (n1-n2)	Rank	OR (95% CI)	p-value^1^
	Cases, N	HPV, n1 (%)	Cases, N	HPV, n2 (%)				
Multiple	187	54 (28.9)	595	78 (13.1)	15.8	-	2.69 (1.81, 4.00)	<0.001
HPV-16	187	97 (51.9)	595	346 (58.2)	-6.3	1	0.77 (0.56, 1.08)	0.150
HPV-18	187	46 (24.6)	595	110 (18.5)	6.1	2	1.43 (0.97, 2.12)	0.075
HPV-45	187	24 (12.8)	595	75 (12.6)	0.2	14	1.02 (0.62, 1.67)	0.900
HPV-56	150	11 (7.3)	555	7 (1.3)	6.0	3	6.18 (0.85, 2.79)	<0.001
HPV-33	150	9 (6.0)	555	31 (5.6)	0.4	13	1.08 (0.51, 2.32)	0.843
HPV-31	150	8 (5.3)	555	12 (2.2)	3.1	4	2.55 (1.02, 6.35)	0.050
HPV-58	150	7 (4.7)	555	9 (1.6)	3.1	4	2.96 (1.08, 8.08)	0.060
HPV-66	53	2 (3.7)	54	1 (1.9)	1.8	7	2.08 (0.70, 23.57)	0.618
HPV-82	53	2 (3.7)	54	1 (1.9)	1.8	7	2.08 (0.70, 23.57)	0.618
HPV-51	150	5 (3.3)	555	3 (0.4)	2.9	6	6.34 (1.52, 26.58)	0.013
HPV-52	150	4 (2.7)	555	14 (2.5)	0.2	14	1.06 (0.34, 3.29)	1.000
HPV-68	97	2 (2.1)	501	2 (0.4)	1.7	9	5.25 (0.73, 37.7)	0.125
HPV-35	150	3 (2.0)	555	22 (4.0)	1.6	10	0.49 (0.15, 1.67)	0.325
HPV-39	150	3 (2.0)	555	4 (0.7)	1.3	11	2.81 (0.61, 12.7)	0.170
HPV-59	150	2 (1.3)	555	3 (0.5)	0.8	12	2.49 (0.41, 15.0)	0.289
HPV-53	37	0 (0.0)	40	0 (0.0)	0.0	16	-	-

HPV, human papillomavirus; HIV, human immunodeficiency virus; Diff, difference; OR, odds ratio; CI, confidence interval; N, number of cases investigated (with adequate information on HPV positivity).

1 Chi-square test.
